# Diagnostic sensitivity of SILVAMP TB-LAM (FujiLAM) point-of-care urine assay for extra-pulmonary tuberculosis in people living with HIV

**DOI:** 10.1183/13993003.01259-2019

**Published:** 2020-02-06

**Authors:** Andrew D. Kerkhoff, Bianca Sossen, Charlotte Schutz, Elena Ivanova Reipold, Andre Trollip, Emmanuel Moreau, Samuel G. Schumacher, Rosie Burton, Amy Ward, Mark P. Nicol, Graeme Meintjes, Claudia M. Denkinger, Tobias Broger

**Affiliations:** 1 Division of HIV, Infectious Diseases and Global Medicine at Zuckerberg San Francisco General Hospital and Trauma Center, Dept of Medicine, University of California, San Francisco, CA, USA; 2 Wellcome Centre for Infectious Diseases Research in Africa, Institute of Infectious Disease and Molecular Medicine, University of Cape Town, Cape Town, South Africa; 3 Dept of Medicine, Faculty of Health Sciences, University of Cape Town, Cape Town, South Africa; 4 FIND, Geneva, Switzerland; 5 Southern African Medical Unit, Médecins sans Frontières, Cape Town, South Africa; 6 Division of Infection and Immunity, School of Biomedical Sciences, University of Western Australia, Perth, Australia; 7 Division of Medical Microbiology, University of Cape Town, Cape Town, South Africa; 8 Division of Tropical Medicine, University of Heidelberg, Heidelberg, Germany; 9 Contributed equally; 10 Contributed equally

## Abstract

Diagnosing tuberculosis (TB) in people living with HIV (PLHIV) remains challenging in part, because of its diversity of clinical manifestations, including high rates of extra-pulmonary and disseminated disease [1]. In particular, disseminated TB, involving multiple organ systems, is associated with high mortality but often presents non-specifically, which may hinder prompt diagnosis [2, 3]. Xpert MTB/RIF (Xpert; Cepheid, Sunnyvale, CA, USA), is currently recommended by the World Health Organization (WHO) as the first line assay for evaluating a subset of extra-pulmonary TB disease (EPTB) manifestations [4].

## Abstract 2

**FujiLAM point-of-care rapid urine test for TB detects a large proportion of pulmonary and traditionally difficult-to-diagnose extra-pulmonary forms of TB in hospitalised patients with advanced HIV** http://bit.ly/36Dl0Eo

To the Editor:

Diagnosing tuberculosis (TB) in people living with HIV (PLHIV) remains challenging in part, because of its diversity of clinical manifestations, including high rates of extra-pulmonary and disseminated disease [1]. In particular, disseminated TB, involving multiple organ systems, is associated with high mortality but often presents non-specifically, which may hinder prompt diagnosis [2, 3]. Xpert MTB/RIF (Xpert; Cepheid, Sunnyvale, CA, USA), is currently recommended by the World Health Organization (WHO) as the first line assay for evaluating a subset of extra-pulmonary TB disease (EPTB) manifestations [4]. To detect specific forms of EPTB, such as pleural TB, TB meningitis or TB lymphadenitis, Xpert may require an invasive sample to be collected, which often limits its use for EPTB detection to hospitals where appropriate equipment is available and invasive sampling can be safely performed. Furthermore, even when concomitant pulmonary disease is present, it can be very difficult to obtain sputum in the sickest HIV patients to submit for Xpert testing [5, 6]. Therefore, an urgent priority for improving TB detection among PLHIV remains the development of rapid, point-of-care (POC) assays that use an easily obtainable clinical specimen, such as urine, and that have good diagnostic accuracy for both pulmonary and EPTB, including disseminated disease [7].

The commercially available Alere Determine TB LAM (AlereLAM; Abbott, Chicago, IL, USA) assay is a rapid, inexpensive, urinary POC TB test [8]. While its use is associated with a mortality benefit in severely ill and immunocompromised PLHIV [9, 10], it has only moderate sensitivity that is limited to patients with low CD4 counts, which has led to limited programmatic uptake [11]. We have previously reported on the Fujifilm SILVAMP TB LAM (FujiLAM; Fujifilm, Tokyo, Japan) POC assay that, similar to AlereLAM, detects the presence of lipoarabinomannan (LAM) in urine [12]. It offers on average 30% improved sensitivity for detecting TB (independent of whether it is PTB or EPTB) compared to AlereLAM across subgroups stratified by CD4 strata, while maintaining high specificity. Here we report the sensitivity of FujiLAM in comparison to AlereLAM specifically for detecting EPTB in the same patient cohorts.

This *post hoc* analysis utilised data from two previously published, prospective cohort studies of adults (>18 years) living with HIV who were admitted to South African district hospitals on the outskirts of Cape Town [13, 14]. Cohort A enrolled patients without a current TB diagnosis regardless of presenting signs or symptoms, and independent of CD4 count [13]. Cohort B enrolled patients with a CD4 count <350 cells·μL^−1^ in whom TB was considered the most likely diagnosis on admission [14]. A third previously published cohort was not included in the present analysis as it excluded patients with exclusively extra-pulmonary TB disease [12]. Informed consent was obtained from patients who had capacity or regained capacity and all study-related activities were approved by the Human Research Ethics Committee of the University of Cape Town.

Patients were systematically evaluated for the presence of TB. Whenever possible, patients provided two sputum samples, a blood sample and a urine sample for mycobacteriology; those unable to produce sputum or urine samples were not excluded from study enrolment. Sputum specimens were tested using smear fluorescence microscopy, mycobacteria growth indicator tube (MGIT) liquid culture (Becton Dickinson, Franklin Lakes, NJ, USA), and Xpert MTB/RIF Version G4. Blood specimens were tested using BACTEC Myco/F Lytic culture (Becton Dickinson, Franklin Lakes, NJ, USA). Sediments from urine specimens were tested using Xpert after centrifugation of 30–40 mL. The routine clinical team obtained additional specimens (sputum and non-sputum) as clinically indicated. FujiLAM and AlereLAM were performed on biobanked urine samples according to manufacturers' instructions and read by two investigators blinded to patient status and all other test results [12]. Microbiologically confirmed TB was defined by the detection of *M. tuberculosis* on any clinical specimen using either culture or Xpert. All patients with microbiologically-confirmed TB were classified into one of three mutually-exclusive groups: pulmonary TB (PTB) (TB detected in sputum only), EPTB (TB detected in extra-pulmonary specimen(s) only), or PTB+EPTB (TB detected in both sputum and at least one extra-pulmonary specimen). The sensitivity (and corresponding 95% confidence intervals) of FujiLAM and AlereLAM was calculated for each form of TB as well as for individual forms of EPTB.

Of 1079 eligible patients, 111 had a TB status that could not be classified, 90 did not have urine samples and six had missing urine results; therefore, 872 patients (420 from cohort A and 659 from cohort B) had complete results and were included in this analysis. The median age was 36 (IQR 30–43) years, 54% were female, the median CD4 count was 84 (IQR 32–188) cells·μL^−1^, and 45% had previously been treated for TB. Among 872 patients, 553 (138 from cohort A and 415 from cohort B) had microbiologically confirmed TB (prevalence 56%) on at least one specimen, 88 (37 from cohort A and 51 from cohort B) had possible TB and 231 (189 from cohort A and 42 from cohort B) had no evidence of TB. Of those with confirmed TB, 126 (23%) out of 553 had PTB, 156 (28%) out of 553 had EPTB, and 271 (49%) out of 553 had both PTB+EPTB. The urine LAM assays performed best in those with PTB+EPTB, with FujiLAM detecting 91% (95% CI 87–94; 246 out of 271) of cases compared with 61% (95% CI 55–67; 165 out of 271) using AlereLAM (figure 1a). In patients with PTB or EPTB only, FujiLAM detected 60% (95% CI 51–69; 76 out of 126) and 67% (95% CI 59–75; 105 out of 156) of cases, respectively, which was compared with 19% (95% CI 12–27; 24 out of 126) and 41% (95% CI 33–49; 64 out of 156), respectively for AlereLAM (figure 1a).

**FIGURE 1:**
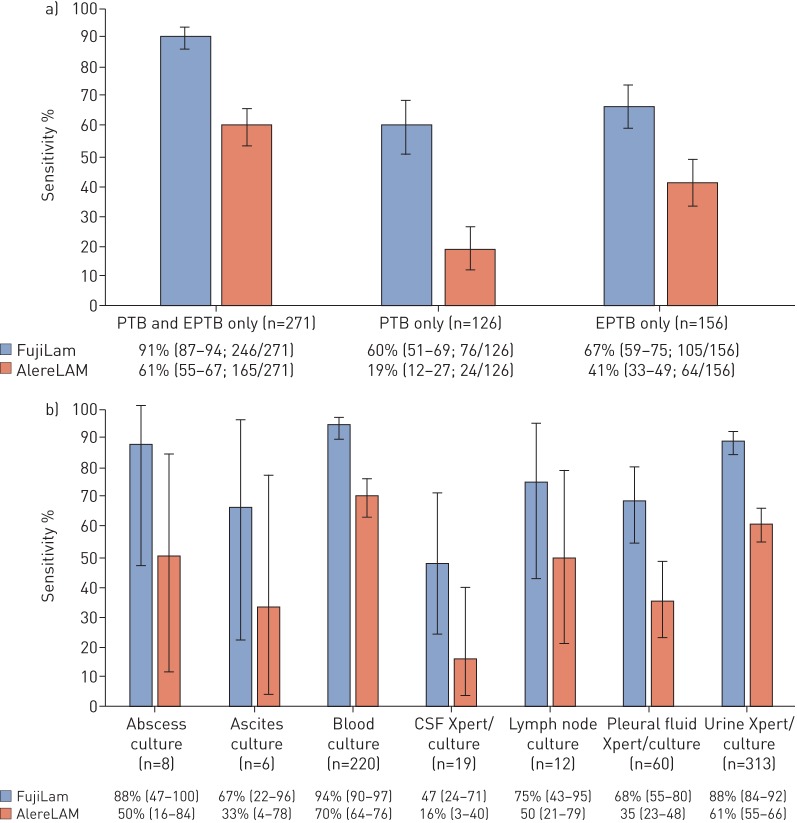
The diagnostic sensitivity of FujiLAM and AlereLAM by (a) type of tuberculosis disease (pulmonary, extra pulmonary or both; n=553), (b) site of disease involvement in patients with confirmed extra-pulmonary tuberculosis (EPTB). Bars represent 95% confidence intervals. The numbers in parenthesis denote 95% confidence intervals. Of note, the same patient may have multiple sites of confirmed disease (*e.g.*, pulmonary, blood, urine, etc). Sputum, blood and urine were obtained from all patients whenever possible and additional specimens were obtained at this discretion of routine medical team, however the ability to produce sputum was not a requirement for study entry. This analysis was limited to among those with both FujiLAM and AlereLAM results available. PTB: pulmonary tuberculosis.

The sensitivity for FujiLAM across different extra-pulmonary forms of TB disease ranged from 47 to 94% as shown in figure 1b. Notably, FujiLAM detected TB in 94% (95% CI 90–97) of patients with TB mycobacteraemia and 88% (95% CI 84–92) of those with TB confirmed by urine Xpert or culture. It also demonstrated moderate sensitivity in patients with microbiologically-confirmed pleural TB (68%; 95% CI 55–80) and with TB meningitis (47%; 95% CI 24–71). AlereLAM's sensitivity ranged from 16 to 70% and performed best in those with TB mycobacteraemia (70%; 95% CI 64–76) and TB confirmed by urine Xpert or culture (61%; 95% CI 55–67).

Overall, FujiLAM showed substantially higher sensitivity over the commercially available AlereLAM, for detecting both pulmonary and extra-pulmonary TB in HIV inpatients. This suggests that FujiLAM may have clinical utility as a first-line test for the rapid detection of TB in HIV patients, independent of disease location. Given that a large proportion of patients with HIV-associated TB have EPTB and a diagnosis may only be possible using a non-sputum sample that may be challenging to obtain, an up-front FujiLAM test could substantially reduce the time to diagnosis. FujiLAM was able to detect TB in 67% (105 out of 156) of patients who could not produce a sputum sample or did not have evidence of pulmonary disease; such patients comprised 28% of the study cohort.

FujiLAM performed best in those with TB mycobacteraemia as well as those with concomitantly positive sputum and non-respiratory cultures, detecting >90% of cases. *Mycobacterium tuberculosis* bacteraemia is one of the most common blood stream infections among PLHIV in sub-Saharan Africa [3] and such patients have an extremely high mortality risk. FujiLAM's excellent performance in those with mycobacteraemia suggests a mechanistic association between disease dissemination and urinary LAM. This finding is supported by our recent study that showed a good association between detection of LAM in urine and serum of TB patients, independent of HIV status [15]. However, even for patients with forms of disease such as pleural TB and TB meningitis that may be compartmentalised, FujiLAM had moderate sensitivity, which could add substantial benefit in these cases. Taken together, these findings suggest that LAM antigenuria is likely indicative of glomerular filtration of circulating LAM (or LAM fragments) in addition to renal TB [16]. Further research, that aims to detect LAM with ultra-sensitive platforms, as well as characterisation of LAM structure in urine, is needed to better understand the mechanisms by which LAM enters the bloodstream and urine. This may help to further refine urine-based diagnostics and catalyse the development of blood-based assays. As the overall load of mycobacteria is expected to be higher in HIV-positive patients, our findings should not be generalised to HIV-negative individuals with EPTB.

Patients were not systematically evaluated for the presence of EPTB beyond mycobacterial blood cultures and urine Xpert; additional systematic sampling (*e.g.* pleural fluid or cerebrospinal fluid) could not be justified as it would require invasive sampling where this was not clinically indicated. Coupled with challenges in universally obtaining clinically indicated samples (*e.g.* sputum), some misclassification of TB category (PTB, EPTB or PTB+EPTB) is likely present. Furthermore, because the overall cohort was severely immunocompromised (increasing the likelihood of disease dissemination) and because those with lower, site-specific mycobacterial burdens (CSF or pleural fluid) may not have been diagnosed by Xpert or culture, the true sensitivity of urinary FujiLAM (and AlereLAM) for localised extra-pulmonary disease is possibly an overestimate and may not be generalisable to all patients with these forms of disease. However, the additional specimens collected by the routine clinical team mirrored common practice in settings with a high-burden HIV-associated TB and followed clinical symptomatology. Finally, we did not evaluate the specificity of FujiLAM for specific disease forms given a lack of systematic EPTB sampling. We have previously reported the cohort-specific specificity as well as the estimated specificity using a Bayesian bivariate random-effects model in three cohorts: using a composite reference standard, the specificity of FujiLAM was 95.7% (95% CI 92.0–98.0%) compared with 98.2% (95% CI 95.7–99.6%) for AlereLAM [12].

In conclusion, our results suggest that the POC FujiLAM has good sensitivity for detecting both pulmonary and extra-pulmonary forms of TB in patients with advanced HIV, in which conventional diagnostics may be slow, require infrastructure and equipment or rely on samples that are difficult to obtain. While appropriate sampling should still be undertaken to allow for drug susceptibility testing, FujiLAM may be an appropriate first microbiological TB investigation for all hospitalised PLHIV, allowing for more rapid initiation of anti-TB therapy.

## Shareable PDF

10.1183/13993003.01259-2019.Shareable1 This one-page PDF can be shared freely online.
Shareable PDF ERJ-01259-2019.Shareable
