# Supplementary material for: Diagnostic sensitivity of SILVAMP TB-LAM (FujiLAM) point-of-care urine assay for extra-pulmonary tuberculosis in people living with HIV
Source: Eur Respir J. 2020 Feb 6;55(2):1901259. doi: 10.1183/13993003.01259-2019 (PMC7002975; doi:10.1183/13993003.01259-2019)
Supplement: Supplementary file 1 [file ERJ-01259-2019.Shareable.pdf]

XML PARSER ERRORS IN "MAINTXT" STREAM

SHAREABLE PDF

ERJerjEuropean Respiratory JournalEur Respir J0903-19361399-3003European Respiratory Society10.1183/13993003.01259-2019ERJ-01259-2019AGORARESEARCH LETTER

Diagnostic sensitivity of SILVAMP TB-LAM (FujiLAM) point-of-care urine assay for extra-pulmonary tuberculosis in people living with HIVA.D. KERKHOFFRESEARCH LETTERDiagnostic sensitivity of SILVAMP TB-LAM assay

KerkhoffAndrewD.[1,9], SossenBianca[2,3,9],SchutzCharlotte[2,3], ReipoldElenaIvanova[4], TrollipAndre[4],MoreauEmmanuel[4],SchumacherSamuelG.[4], BurtonRosie[5],WardAmy[2,3],NicolMarkP.[6,7], MeintjesGraeme[2,3], DenkingerClaudiaM.[4,8,10], BrogerTobias[4,10],

1Division of HIV, Infectious Diseases and Global Medicine at Zuckerberg San Francisco General Hospital and Trauma Center, Dept of Medicine, University of California, San Francisco, CA, USA. 2Wellcome Centre for Infectious Diseases Research in Africa, Institute of Infectious Disease and Molecular Medicine, University of Cape Town, Cape Town, South Africa. 3Dept of Medicine, Faculty of Health Sciences, University of Cape Town, Cape Town, South Africa. 4FIND, Geneva, Switzerland. 5Southern African Medical Unit, Médecins sans Frontières, Cape Town, South Africa. 6Division of Infection and Immunity, School of Biomedical Sciences, University of Western Australia, Perth, Australia. 7Division of Medical Microbiology, University of Cape Town, Cape Town, South Africa. 8Division of Tropical Medicine, University of Heidelberg, Heidelberg, Germany. 9Contributed equally. 10Contributed equally.

Correspondence: Andrew D. Kerkhoff, University of California, San Francisco, Division of HIV, Infectious Diseases and Global Medicine, 1001 Potrero Avenue, San Francisco, California, 94110, USA. E-mail: andrew.kerkhoff@ucsf.edu2020202055024062019181020192020

To the Editor:

Diagnosing tuberculosis (TB) in people living with HIV (PLHIV) remains challenging in part, because of its diversity of clinical manifestations, including high rates of extra-pulmonary and disseminated disease [1]. In particular, disseminated TB, involving multiple organ systems, is associated with high mortality but often presents non-specifically, which may hinder prompt diagnosis [2, 3]. Xpert MTB/RIF (Xpert; Cepheid, Sunnyvale, CA, USA), is currently recommended by the World Health Organization (WHO) as the first line assay for evaluating a subset of extra-pulmonary TB disease (EPTB) manifestations [4]. To detect specific forms of EPTB, such as pleural TB, TB meningitis or TB lymphadenitis, Xpert may require an invasive sample to be collected, which often limits its use for EPTB detection to hospitals where appropriate equipment is available and invasive sampling can be safely performed. Furthermore, even when concomitant pulmonary disease is present, it can be very difficult to obtain sputum in the sickest HIV patients to submit for Xpert testing [5, 6]. Therefore, an urgent priority for improving TB detection among PLHIV remains the development of rapid, point-of-care (POC) assays that use an easily obtainable clinical specimen, such as urine, and that have good diagnostic accuracy for both pulmonary and EPTB, including disseminated disease [7].

Copyright ©ERS 2020. This version is distributed under the terms of the Creative Commons Attribution Licence 4.0.
